# Root exudates and rhizosphere microbiota in responding to long-term continuous cropping of tobacco

**DOI:** 10.1038/s41598-024-61291-0

**Published:** 2024-05-17

**Authors:** Abo Li, Keke Jin, YuZhen Zhang, Xiaopeng Deng, Yi Chen, Xiaomeng Wei, Binbin Hu, Yonglei Jiang

**Affiliations:** 1 Yunnan Academy of Tobacco Agricultural Sciences, Kunming, 650021 China; 2 College of Life Sciences, Northwest A&F University, Yangling, 712100 Shaanxi China; 3 Horticulture Institute, Ningxia Academy of Agriculture and Forestry Sciences, Yinchuan, 750002 China; 4 College of Natural Resources and Environment, Northwest A&F University, Shaanxi, 712100 China; 5 Qingdao Agricultural University, Nanjing, 210095 China

**Keywords:** Continuous cropping, Root exudate, Microbial community, Co-occurrence network, Suppressive soil, Agroecology, Microbial ecology, Plant stress responses

## Abstract

Soil sickness a severe problem in tobacco production, leading to soil-borne diseases and reduce in tobacco yield. This occurs as a result of the interaction between root exudates and rhizosphere microorganisms, which is however, little studied until now. By combining the field investigation and pot experiment, we found the output yield consistently decreased during the first 10 years of continuous cropping in a tobacco field, but increased at the 15th year (15Y). The root exudate and rhizosphere bacterial community was further analyzed to reveal the underlying mechanism of the suppressive soil formation. Root exudate of 15Y tobacco enriched in amino acids and derivatives, while depleted in the typical autotoxins including phenolic acids and alkaloids. This was correlated to the low microbial diversity in 15Y, but also the changes in community composition and topological properties of the co-occurrence network. Especially, the reduced autotoxins were associated with low Actinobacteria abundance, low network complexity and high network modularity, which significantly correlated with the recovered output yield in 15Y. This study revealed the coevolution of rhizosphere microbiota and root exudate as the soil domesticated by continuous cropping of tobacco, and indicated a potential role of the autotoxins and theirs effect on the microbial community in the formation of suppressive soil.

## Introduction

Continuous cropping is one of the greatest threats for sustainable agriculture, because of the limited arable land and increasing populations, which is caused as a results of soil degradation^1–3^. Decrease in soil fertility is generally found in continuous cropping field. This is caused by the selective uptake of soil nutrients by the mono plant, but also a result of the imbalance in soil microbial diversity and functions^4^. Another important attributor of continuous cropping is the autotoxic allelopathy^5^. As a special form of allelopathy, many plants produce allelochemicals through root exudation to outcompete the future generations for resource and space. These autotoxins accumulate in continuous cropping soil and strong impair the crop production^5,6^. Tobacco is widely used as a model organism, and is one of the most important industrial crops across the world. Tobacco is more susceptive to soil sickness in comparation to other crops, however is increasingly planted in continuous-cropping pattern due to the rising demand for food production^6^. Even under optimal field management, continuous cropping can strongly inhibit the plant seedling and development, and on average, reducing the tobacco yield by 20–100%^7,8^. Despite, recent studies suggested that plants can recover from the obstacle after long-term continuous cropping. The phenomenon is also observed for tobacco, but the underlying mechanism remains little studied^9^.

Tobacco is distinct from other crops by the vigorous root exudation of biological active secondary metabolites^10^. Previous experiment found that the tobacco growth was directly inhibited by the leachate of soil with continuous cropping, which was mainly attributed to the allelopathic autotoxicity^11^. In a soil continuously planted by tobacco for 12 years, the researchers identified eight autotoxins, while only one was found in the control^12^. By analyzing the root exudates of tobacco, a wide range of autotoxic substances were recognized^13^. These substances are typically small molecular aromatic compounds, organic acids and alkaloids, generally containing C=O, –OH and C=C^14,15^. The chemical structure makes them decomposition-recalcitrant and possible to accumulate in soil. The composition and amount of autotoxic root exudate varied with the duration of continuous cropping, and is closely related to the intensity of soil sickness. The concentrate of Sanqi ginseng in the soil extract increased with time during a three-year monoculture of Panax notoginseng^16^. However, the accumulation rate of phenolic acid was significantly lower after 10–14 round of continuous cultivation of cucumber than the early stage (0–2 round)^17^. Figuring the temporal dynamic of root exudate is important to understand and alleviate the soil sickness. Nevertheless, this is merely studied for tobacco.

As the most active component of soil, rhizosphere microorganisms affect the soil physiochemical properties and functional processes, promote the root uptake of nutrients, play as pathogens and also can inhibit the soil-borne disease^18,19^. The microbial community of rhizosphere soil is important to both the occurrence and alleviate of soil sickness, therefore has attracted particle attentions^20,21^. Compared with the rotation cropping, continuous cultivation of tobacco was found to concentrate the Acidobacteria, likely a result of soil acidification^22^. Since continuous cropping reduced the soil fertility, the life strategy of rhizosphere microbiota shifted from r to K^8^. Decrease in soil enzyme activities were also reported, which indicated the degradation in soil ecological function^23^. Root exudates act as a bridge connecting plant and rhizosphere microbiota^24^. They provide carbon and energy to cluster microorganisms in a narrow volume of soil close to the root, making rhizosphere a hotspot for plant–microbe-soil interaction. Some of the root exudates are signal chemicals, which recruit certain microbial taxa while repel the others^25^. More importantly, rhizosphere microorganisms decompose the autotoxins to directly mitigate the damage of continuous cropping^5^. Accumulation of benefit microorganisms by root exudate is thought to be an important way to spontaneously overcome the soil sickness in field^5^. However, the relationship between root exudates and rhizosphere microorganisms of tobacco is little known.

In comparison to the mature plant, the seed germinant and seedling development more susceptible to the continuous cropping^26,27^. In this study, we found 15 years mono cultivation of tobacco led to high production yield than short duration of continuous cropping in a field experiment. Based on this phenomenon, we transplanted tobacco seedlings in the soils collected from fields mono-cultivated tobacco for 2–15 years. The root exudates and rhizosphere bacterial community was analyzed after 30 days of incubation, and their relationships to the tobacco yield in the field were addressed. The results help to understand the generation of suppressive soil and to control the soil sickness.

## Materials and methods

### Field experiment and soil collection

Soils used in this study were collected from the long-term tobacco cropping experimental filed located in Midu county, Dali Prefecture, Yunnan Province, China (25° 40ʹ N, 100° 45ʹ E, elevation 1707 m). The climate in this area was low latitude plateau subtropical monsoon, with the mean annual precipitation and mean annual temperature of 1066.5 mm and 14.8 °C, respectively. The field was planted by garlic before the experiment. Thirty separated plots (100 × 6 m) were settled in 2007, 3 of which were randomly chosen to transformed to tobacco plant in 2007, 2012, 2014, 2017 and 2020, respectively. Thus, in 2022 when this study applied, we got soils continuously cultivated tobacco for 2, 5, 8, 10 and 15 years (2Y, 5Y, 8Y, 10Y and 15Y). Five topsoil cores (0–20 cm) were randomly collected from a single plot, and 15 soil cores from the same treatment (continuous cropping duration) were well mixed to make one sample (~ 6 kg) in April 2022, 2 days before the basal fertilizer application. The soils were translated to the laboratory for the follow pot experiment. While in the field, Tobacco seedlings was transplanted at late April, with the variety of Honghuadajinyuan, which is cultivated by Yunnan Academy of Tobacco Agricultural Sciences (Kunming, China). The basal fertilizer was applied 5 days before transplanting, at the rate of 60 kg N ha^−1^, 60 kg P_2_O_5_ ha^−1^ and 144 kg K_2_O ha^−1^. Additionally, 15 kg N ha^−1^, 15 kg P_2_O_5_ ha^−1^ and 36 kg K_2_O ha^−1^ was applied every 20 days during the first 60 days after transplanting. The tobacco yield and output value were calculated according to National standard of the People’s Republic of China: flue-cured tobacco (GB2635-1992). All methods were performed in accordance with the relevant guidelines and regulations.

### Pot experiment

The fresh soils were sieved through a 4 mm mesh after plant residues and stones removed by hand. One kg (dry weight) soil was packed into a rhizobox with one side removable for the follow pot experiment. Four replicates were prepared for each treatment (i.e., the continuous cropping duration). Tobacco seeds were germinated and cultivated for 60 days using the float breeding method. The variety was same as that in the field. One tobacco seedling was transplanted to each rhizobox, and cultivated in a walk-in phytotron at 30 °C and 12/12 h light/dark cycle. The rhizobox was 60° inclined during the whole cultivation to the root grow along the removable side, which would facilitate the collection of root exudate.

### Root exudate collection and analysis

The root exudate was collected after 30 days cultivation using the Hogland medium vas following: the rizhobox was opened from the removable side. The root was carefully moved from the soil surface and washed using sterilized ddH2O until the flow-down liquid was clear without visible soil particles. Two–three clean roots were immersed in the 20 ml Hogland medium and cultivated for 6 h to collect the root exudate. The solutions were frozen-dried at − 45 °C and re-dissolved using 1 ml ddH2O. The soil surface was protected using a polyethylene film to prevent rapid water evaporation and drought stress for tobacco plants after one side of the rhizobox removed. We failed to transplanting of tobacco seedling on the soil of 10Y due to the strong soil sickness, thus root exudate was only collected for 2Y, 5Y, 8Y and 15Y. Since one of the replicates of 8Y was polluted by soil particles during the collection, we randomly discharged one replicate from each treatment to keep sample balance. As a result, 12 root exudates were seed to Wuhan Metaware Biotechnology Co., Ltd. (www.metaWare.cn; Wuhan, China) for metabonomic analysis using LC–MS. The analytical conditions were as follows: UPLC column, Waters ACQUITY UPLC HSS T3 C18 (1.8 μm, 2.1 mm × 100 mm); column temperature, 40 °C; flow rate, 0.4 mL/min; injection volume, 2 μL; solvent system, water (0.1% formic acid): acetonitrile (0.1% formic acid); gradient program, 95:5 V/V at 0 min, 10:90 V/V at 11.0 min, 10:90 V/V at 12.0 min, 95:5 V/V at 12.1 min, 95:5 V/V at 14.0 min.

### Soil sampling, DNA extraction and high-throughput sequencing

The whole plant was removed from the rhizobox after root exudate collection, and the rhizosphere soil was collected by shaking the root by hand. For 10Y, soil was collected from the central of the rhizobox. Thus, 20 soils were sampled in total. Soil from each rhizobox was well mixed and 0.5 g fresh soil was used for DNA extraction immediately. The DNA was extracted using Qiagen PowerSoil DNA kit following the manufacturer’s certificate. The DNA quality and quantity were checked on a 1.5% agarose gel and Nanodrop 100 UV–Vis spectrophotometer (Thermo Scientific, Wilmington, United States), respectively, and stored at − 20 °C for further analysis. The bacterial 16S rRNA gene was amplified using the primer pair 338F/806R (5ʹ-ACTCCTACGGGAGCAGCAGC-3′/5′-GGACTACHVGGGTWTCTAAT-3′). The PCR amplication was performed using the following thermal contdition: 98 °C for 1 min, followed by 30 cycles at 98 °C for 10 s, 52 °C for 30 s, and 72 °C for 30 s, and a final extension at 72 °C for 5 min. The PCR mixture contained 10 μl 2 × Premix PCR Ex Taq (Takara, Japan), 1 μl DNA templete (20 ng μl^−1^), 0.2 μl of each primer and 8.6 μl sterilized ddH2O. The PCR production was gel purified and sequenced on Illunima NovaSeq platform using the pair ends method (2 × 250 bp) in Novogene Co., Ltd. (Beijing, China).

### Sequence processing and statistical analysis

The paired-end sequences were merged by FLASH and proposed in Qiime2 platform (https://view.qiime2.org). Fastq sequences were filtered and demultiplexed using dada2. Representative sequences were aligned using MAFFT, and the taxonomies of the representative sequences were assigned using QIIME feature-classifier. Raw sequences were deposited in the NCBI Sequence Read Archive with the accession number of PRJNA1035762 (http://www.ncbi.nlm.nih.gov/bioproject/1035762).

Further analysis was performed using R.4.2.1 (https://www.r-project.org/). All the samples were rarefied to the sequencing depth of 27,603 (the minimum sequencing depth of the samples) rarefy_even_depth function in phyloseq package. Uisng the estimate_richness function in phyloseq, we calculated the OTU richness and Shannon Shannon diversity.

In this study we constructed the co-occurrence network of analyze the complex relationship in microbial community (microbial network) and the relationship between microbial OTUs and root exudates (microbe-exudate network) using the *graph_from_adjacADcy_matrix* function in the *igraph* package. Before constructing the networks, OTUs with relative abundance lower than 0.01% were filtered, and the spearman correlation between remained OTUs or between the OTUs and root exudates were calculated using the *rcorr* function in *Hmisc*. Based on the random matrix theory using *rm.get. threshold* function in *RMThreshold*, the cutoff of 0.72 and 0.86 was chosen for the microbial network and microbe-exudate network, respectively (*p* < 0.05)^28^. To calculate the network topological properties of the microbial community in each sample, we first constructed a global microbial network using all samples and then extracted the subnetwork of each sample from the global network based on the vertex. The *igraph* package was used to calculated the network features including edge number, node number, average degree, positive edge ratio, transitivity betweenness centralization and modularity. In microbe-exudate network, the links between OTUs and between root exudates were removed, which remained only the links between OTUs and root exudates. The Gephi 0.9.7 (https://gephi.org/) was used to visualize the networks.

ANOVA and LSD.test were performed using the *aov* and lsd.test functions, respectively, to check the significant difference among the continuous-cropping time. And Wilcoxon test were used to check the significant difference in the relative abundance of root exudate components and bacterial phyla among the continuous-cropping time using *wilcox.test* function. The relationships between root exudates, microbial community properties and tobacco yield/output value were calculated by Spearman correlation using the rcorr function in *Hmisc.*

## Results

### Tobacco yield and output value

**Figure 1:**
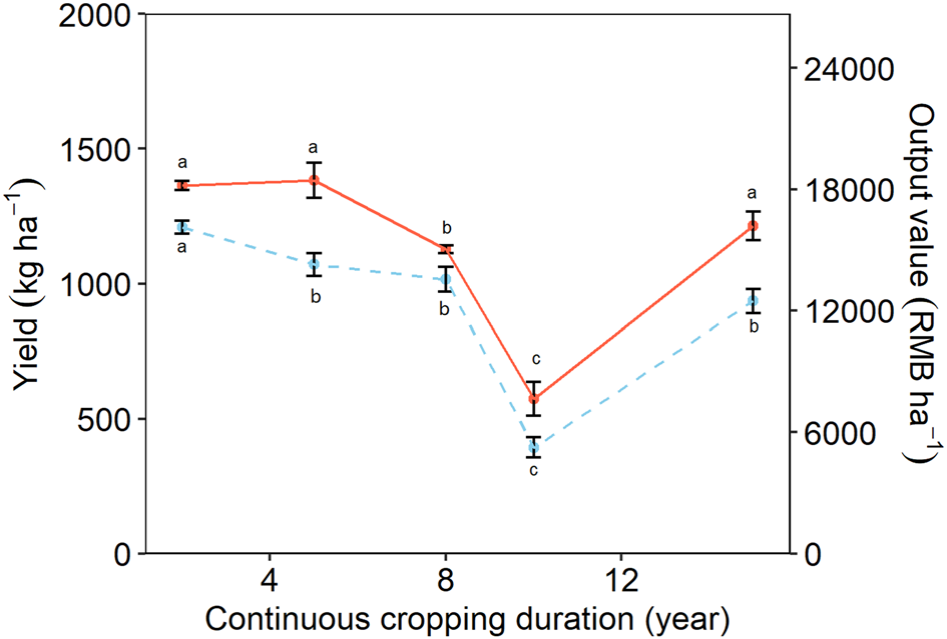
Effect of continuous cropping on the tobacco output yield and value.

The tobacco yield was similar after 2 and 5 years of continuous cropping, and significantly decrease from 5 to 10 years (Fig. 1). After 10 years continuous cropping, the yield was as low as 574.3 kg ha^−1^, which was 42% of that in the 2nd year (p < 0.05). However, increase in the yield was observed in the 15th year (1214.0 kg ha^−1^), which reached the level comparable to that in 2nd and 5th year. Despite 5-year continuous cropping did not significantly affect the tobacco yield in comparison to the 2nd year, the output value decreased by 12% due to the low leaf quality (Fig. 1). Similar to the yield, output value was lowest in the 10th year, and recovered from 10 to 15 years. Nevertheless, we found 23% low output value after 15 years of continuous cropping than the 2nd years. Despite the increasing availability of total P and available N, P and K (Table S1), we found no significant relationship between the soil nutrients and tobacco yield or the output value (Table S2), probably due to the high nutrient content and availability throughout the experiment. Therefore, the soil nutrients were not included in the following analysis.

### Root exudate metabolomics

**Figure 2:**
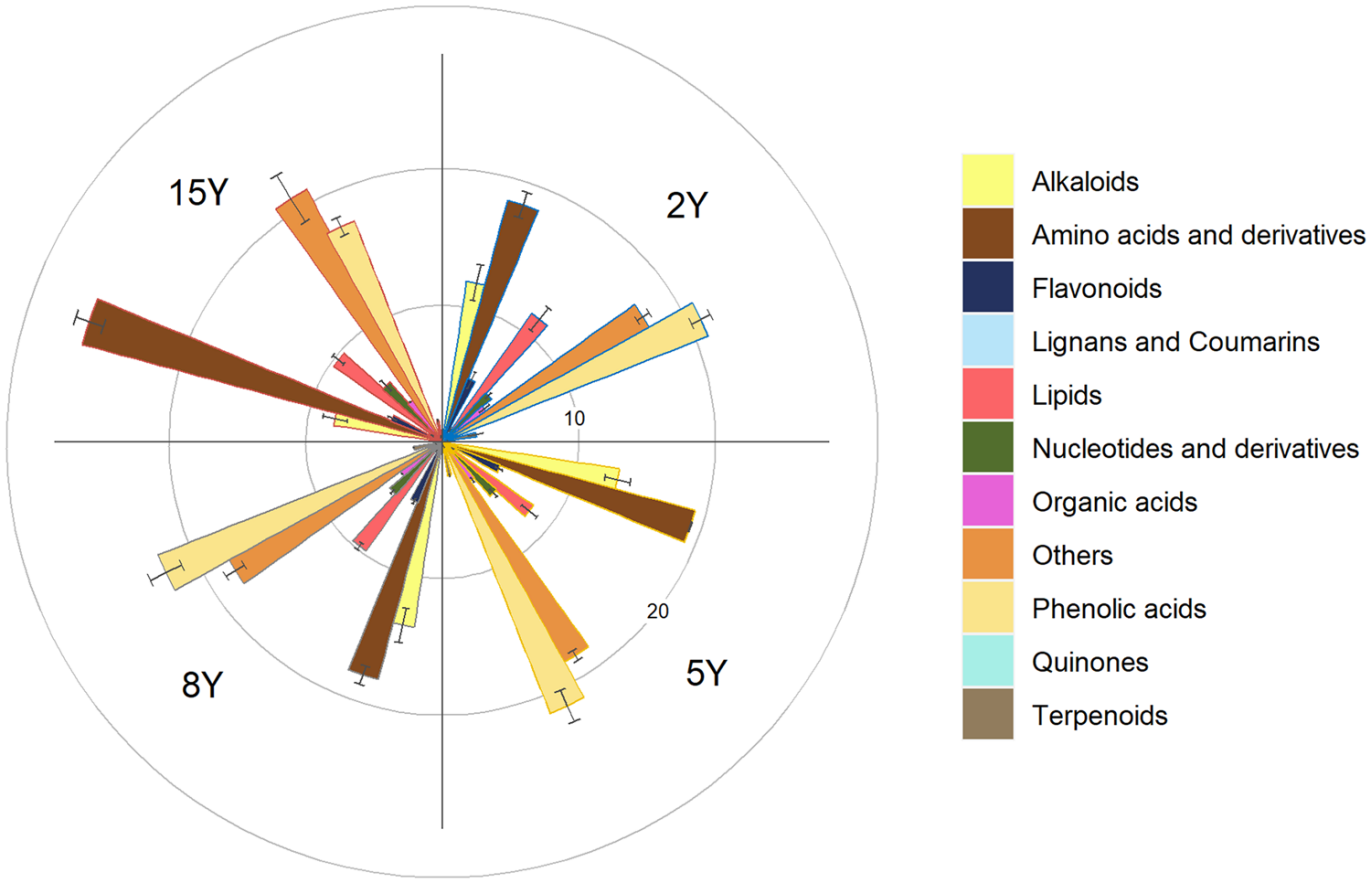
Relative abundance of different metabolites in the root exudate from tobacco plant cultivated in the soils with 2-, 5-, 8- and 15- years (2Y, 5Y, 8Y and 15Y) of continuous cropping.

As shown in the heatmap (Fig. S1), the overall composition of root exudate was little affected by the continuous cropping duration in the first 8 years, but strongly changed after 15 years. The most abundant metabolites in the root exudates from 2Y, 5Y and 8Y was phenolic acids, with the relative abundance increased from 20.9% in 2Y to 22.4% in 8Y (Fig. 2). While in 15Y, the value decreased to 17.4% (p < 0.05). Similarly, the relative abundance of alkaloids also increased from 2nd to 8th year, but decreased in the 15th year (p < 0.05). Other metabolites significantly affected by the continuous cropping duration including lipids, terpenoids and amino acids and derivatives, among which the first two components decreased while the later one increased in the 15 year (p < 0.05). After 15 years of continuous cropping, the most abundant root exudate was amino acids and derivatives, with the relative abundance of 27.3%.

### Soil microbial community and the relationship to root exudates

**Figure 3:**
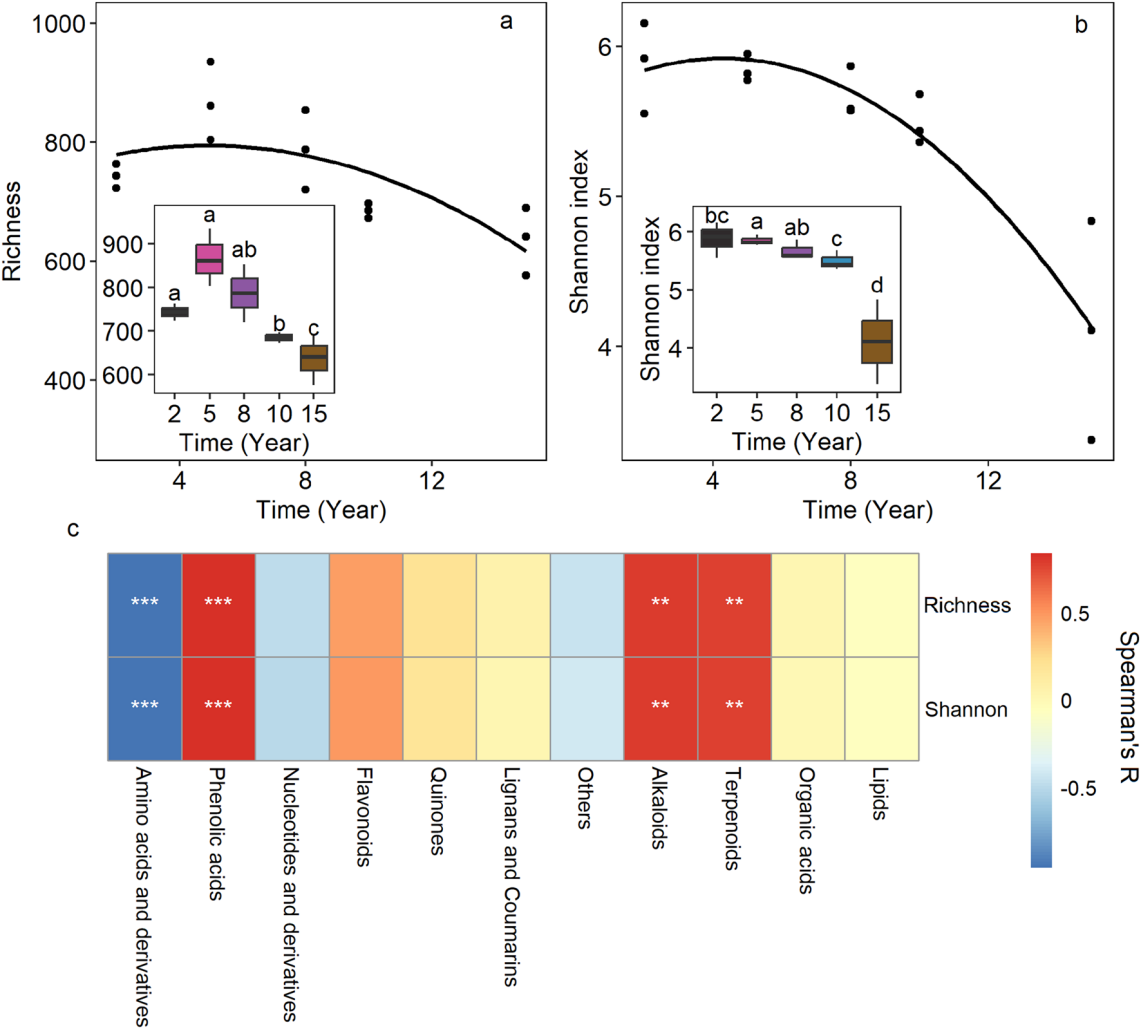
Effect of long-term continuous cropping on the microbial diversity indices (**a**, **b**) and their relationships to the key root exudates (**c**).

The OTU richness and Shannon diversity of bacterial community increased from 2 to 5 years of continuous cropping, but significant difference was only observed for the latter (Fig. 3a,b). Whereas, both the diversity index decreased in further continuous cropping. After 15 years, the richness and Shannon index were 625.3 and 4.1, respectively, which was 85.4% and 69.9% of that in 2Y. The dynamic of soil bacterial diversity as affected by the continuous cropping duration well fitted to unimodal function, with the R2 of 0.856 (*p* < 0.001) and 0.820 (*p* < 0.001) for OTU richness and Shannon index, respectively. We analyzed the relationship between the components of root exudate and bacterial diversity (Fig. 3c). The results indicated that all the metabolites significantly affected by the continuous cropping duration except lipids also significantly correlated with the OTU richness and Shannon index. Negative correlation was found for Amino acids and derivatives, while positive correlation was found for the other 3 components.

**Figure 4:**
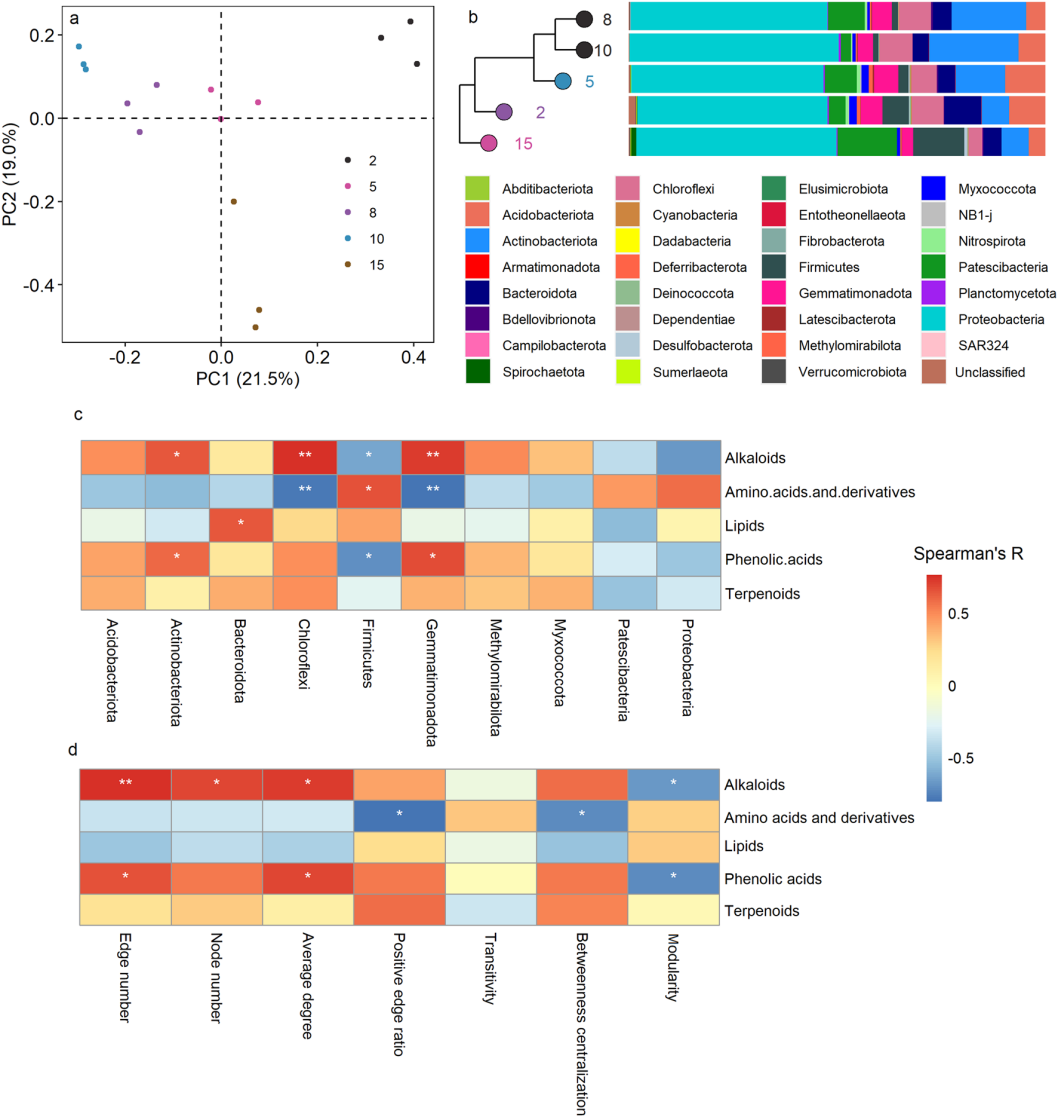
Effect of long-term continuous cropping on microbial community composition revealed by PCoA analysis (**a**), the community composition at phylum level (**b**), and the correlation of dominate phylum (**c**) and co-occurrence network properties (**d**) to the key root exudates.

The bacterial community composition significantly shifted as the duration of continuous cropping increasing (Fig. 4). Despite, the difference among 8Y and 10Y was not significant (Fig. 4a). There were 14 genera with the relative abundance higher than 1%, including Stenotrophomonas (7.9%), Streptomyces (7.5%), Lysobacter (3.5%), etc. (Fig. S2a). However, all these genera were loosely correlated with the key root exudates (Fig. S2b). The most abundant bacteria were Proteobacteria in all treatment, but their relative abundance was little affected by the continuous cropping duration (Fig. 4b). We analyzed the response of top 10 abundant phyla to continuous cropping duration (Fig. 4b). In 8Y and 10Y, we found significantly higher Actinobacteria, while lower Firmicutes than 2Y and 15Y (*p* < 0.05). The other phyla significantly affected by the continuous cropping duration including Bacteroidota, Acidobacteriota, Chloroflexi, Gemmatimonadota, Methylomirabilota, Myxococcota and Patescibacteria. High abundant in 2Y than 15Y was observed for all these phyla except Patescibacteria. Half of the top 10 phyla significantly correlated to at least one of the key root exudates (components significantly affected by continuous cropping), including Actinobacteria, Bacteroidota, Chloroflexi, Firmicutes and Gemmatimonadota (Fig. 4c). Alkaloids and phenolic acids positively correlated with Actinobacteria and Gemmatimonadota while negatively correlated with Firmicutes. Amino acid and derivatives positively correlated with Firmicutes but negatively correlated with Chloroflexi and Gemmatimonadota. Additionally, lipids and alkaloids positively correlated with Bacteroidota and Chloroflexi, respectively.

Relationships between the key root exudates and the topological parameters of the bacterial co-occurrence network were also analyzed (Fig. 4d). The results suggested positive correlations of alkaloids and phenolic acids to the network complexity (indicated by the number of nodes and edges, and the average degree), while negative correlations to the modularity (p < 0.05). Moreover, the amino acids and the derivatives negatively correlated to the network tightness (indicated by the betweenness centralization) and cooperation intensity (indicated by the ratio of positive edges) (p < 0.05).

**Figure 5:**
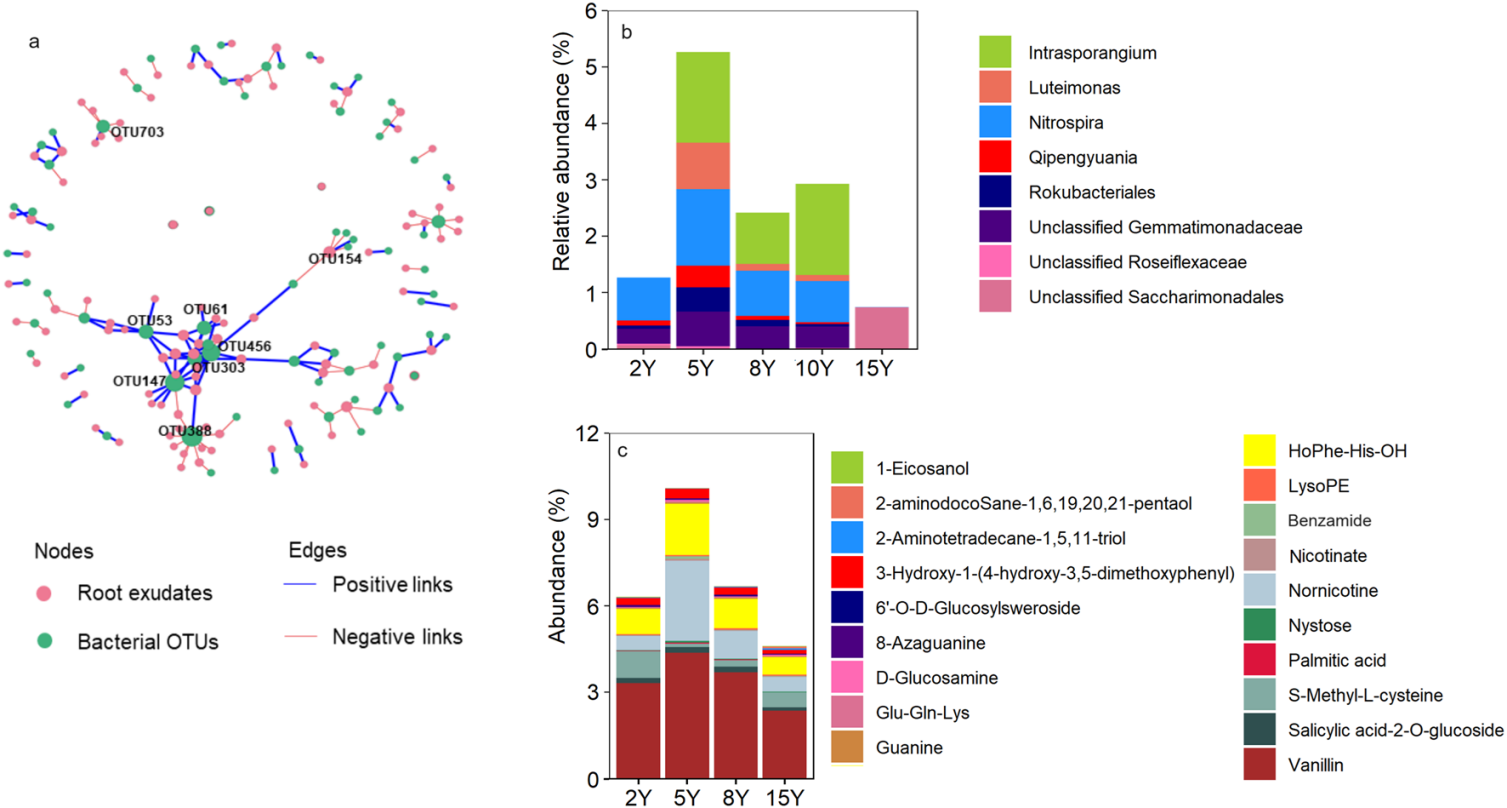
Network showing the relationship of microbial OTUs to the root exudate compounds and the effect of long-term continuous cropping on the abundance of key OTUs and key compounds in the network.

We further analyzed the relationships of individual bacterial OTUs and root exudate compounds (Fig. 5). The network showed 111 root exudates significantly correlated with 88 OTUs (Fig. 5a). We found 19 root exudate compounds with network degree higher than 2, among which only 2 amino acid derivatives (S-Methyl-l-cysteine, Glu-Gln-Lys) and 1 lipid (2-Aminotetradecane-1,5,11-triol) significantly enriched in Y15 (Fig. 5b). There were 8 OTUs with the network degree higher than 5. These OTUs in summary made 68 links to 19 root exudates, accounting for 38.6% of the total links in the network. Among these OTUs, only OTU703 (Unclassified Roseiflexaceae) significantly enriched at Y15, which positively correlated with the amino acids and derivatives (S-Methyl-l-cysteine) while negatively correlated with the phenolic acids (Benzyl β-primeveroside and 3,4-dihydroxyphenyl) and alkaloids (Isopelletierine) (Fig. 5c). All the other key OTUs mainly positively linked to the root exudates (mainly phenolic acids and alkaloids) except OTU388 (*Qipengyuania*) which mainly showed negative relationships to the saccharides and lipids (Fig. 5a).

### Relationship of microbial community to tobacco output

**Figure 6:**
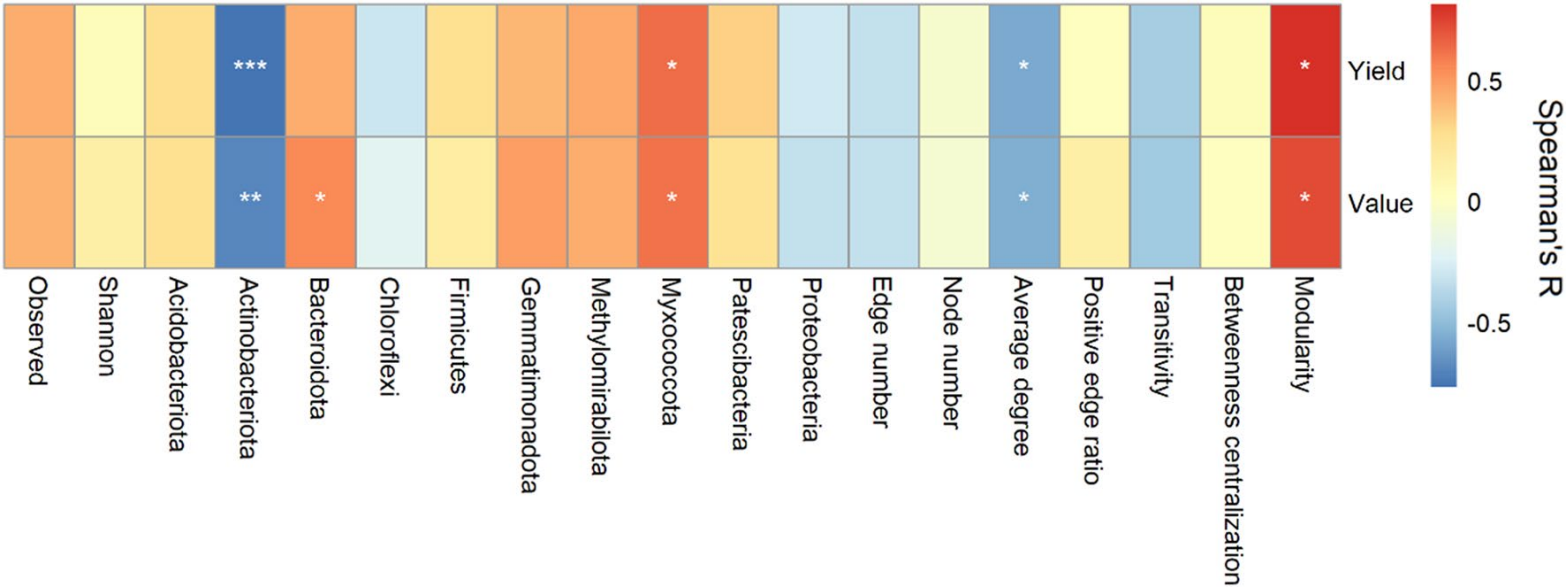
Relationship between soil microbial community traits to tobacco yield and output value.

We found no relationship of the tobacco yield and output value to the relative abundance of all root exudate classes (data not show). However, significant correlations of bacterial community properties to both tobacco yield and output value were detected (Fig. 6). Both the yield and output value negatively correlated to the relative abundance of Actinobacteria (*p* < 0.01) and average degree of the co-occurrence network (*p* < 0.05) but positively correlated to Myxococcota and network modularity (*p* < 0.05). Bacteroidota were also positively correlated to the output value (*p* < 0.05). Nevertheless, the relationships of bacterial diversity to tobacco yield and output value were weak.

## Discussion

By quantifying the leaf yield and output value, this study found increasing soil sickness in the first 10 years mono cultivation of tobacco, which was mitigated when the continuous cropping duration prolonged to 15 years. The results indicated the generation of suppressive soil after 15-year continuous cropping. Based on liquid chromatography tandem mass spectrometry and high-throughput sequencing, we explored the dynamics of tobacco soil bacterial community and metabolomics during long-term continuous cropping. Their relationships to tobacco yield and output value were analyzed. The results help to understand the produce and alleviation mechanisms of soil sickness in tobacco field.

The content of tobacco root exudates was different during different years of continuous cropping (Fig. S1 and Fig. 2). This may be the result of tobacco roots adapting to changing soil environments^24^. Several secondary metabolites were found in the root exudate, such as phenolic acids, alkaloids, terpenes and ketones, which have been recognized as the major autotoxins for many plants^5^. The most abundant compound in our tobacco root exudate were phenolic acids. Phenolic acids are ability to scavenge reactive oxygen species and produced as antioxidant by many plants, especially under environmental stress^29,30^. They would stimulate the plant growth and productivity at low concentration^29,31^. However, high concentration of phenolic acids strongly inhibit the growth of radicle and seedling development^11,31^). The proportion of phenolic acids in the root exudate increased from 2 to 8 years of continuous cropping and may contributed to the failed planting of tobacco in the in ten-year continuous cropping soil. We found significantly lower phenolic acid in the soil mono-cultivated tobacco for 15 year, which might be an important attribution to the reduced soil sickness.

Another major group of root exudate component was amino acids and the derivates, whose proportion little changed from 2 to 8Y, but increased by more than 40% in 15Y. Amino acids are applied to the agricultural soil as new functional organic fertilizers in recent years^32^. First, they are high quality carbon and energy source for bacteria. As most soil is limited in organic matters, they help to construct and enhance the rhizosphere hotspot of microbial processes^33^. Despite plants and microbes prefer to use mineral nitrogen, they also directly uptake small molecular organic nitrogen, mainly amino acids^34,35^. They can work as implement nitrogen source, but more frequently growth promotors. In previous studies, amino acids were found to increase biomass synthesis, promote the flavor and improve the crop yields^32,36,37^. In the treatment 15Y, amino acid over phenolic acid dominated the root exudate components, which might contribute to the increased tobacco yield in comparison to 10Y. In the previous study, removal of amino acids from the root exudate increased the antibacterial effects while reduced the antifungal effects^33^. Considering the flourish of fungal community and serious fungal diseases in continuous cropping, abundant amino acid in 15Y might also benefit the tobacco by inhibiting fungal growth. Amino acids are one of the biggest components of plant carbon input to soil, however is little studied for tobacco^37^. This study suggested its potential importance in alleviating the soil sickness of tobacco.

Soil microorganisms in complex microbial communities participate in the regulation of soil nutrient cycling, affecting soil nutrients, plant growth and ecosystem sustainable development^38^. In the past decades, beneficial bacteria are found to inhibit the pathogens by competing for ecological niches, secreting antibiotics and other pathways, thus alleviate the harm of continuous cultivation^39,40^. Plant root exudates have been shown to shape the soil microbial community in the rhizosphere^25,41^. Rhizosphere microbial community respond to these plant-derived metabolites, which in turn feedback on the soil and plant properties^25,38^. Continuous cropping is well known to alter the soil microbial community^5,20,42^, which was also found in our study (Figs. 3 and 4). Such effect on one hand leads to prevalence of soil-borne pathogens, while on the other hand can re-structure the rhizosphere microbiota to disease suppressive under property management^43,44^.

Similar to our result (Fig. 3), Xiong et al. found that soil spontaneously overcoming the continuous cropping showed low bacterial diversity^45^. This was associated with the high amino acids and derivatives, while low phenolic acids, alkaloids and terpenoids (Fig. 3b). As discussed above, these metabolites were among the root exudates most sensitive to continuous cropping of tobacco, thus were possible to play a role in shaping the microbial diversity. In comparison to the other three compounds, amino acids are more readily decomposable. As reported by Hobbie and Hobbie, soil microorganisms can quickly use the newly produced amino acids within minutes and hours^46^. Previous studies suggested that most bacteria can use amino acids to support their growth. However, bacteria differed in the ability to use amino acidic metabolites. Using the 13C-PLFA method, several studies demonstrated that amino acids and the derivations added to soil were most and fastest enriched in gram-positive bacteria^47^. Consistently, the amino acids and derivations were positive correlated with Firmicutes in this study. Firmicutes are among the fastest growing bacteria with up to 12 copies of ribosomal RNA gene in the genome. They would largely consume the living space and nutrient when benefitted from the amino acidic metabolites, which might contribute to the decrease in bacterial diversity in 15Y. As expected, more amino acids and derivations were associated with less close and more antagonistic relationship among bacterial species (indicated by lower betweenness centralization and positive edge ratio of the co-occurrence network) (Fig. 4d). According to Jin et al., addition of amino acidic (succinic and glutamic acid) led to lowest bacterial diversity in the rhizosphere of cucumber, which was higher when treated with phenolic acids (p-hydroxybenzoic acid and p-coumaric acid)^48^. Similar results were observed in our study, likely indicated weaker filter effect of phenolic acids than amino acid. The results were likely because phenolic acids were chemical recalcitrant, and would lead to little substrate preference of microorganisms over the native soil organic matters. This situation might also suitable for alkaloids and terpenoids due to their stable molecular structure. All the bacterial phyla significantly correlated with these metabolites were know by the capacity to use complex recalcitrant organic matters, which likely supported our above hypothesis. Interspecies cooperation is frequently required to decompose complex substrate^49^. This is consistent to the complex and low-modular network when root prefer to output carbon in the forms of phenolic acids and alkaloids (Fig. 4d). Nevertheless, tobacco yield and output value were benefited from the modularization of microbial community (Fig. 5). Different modules of microbial network are suggested to execute different ecological functions^50^. Higher modularization might indicate higher functional diversity, which were found crucial for suppressive soil^51,52^. However, among the bacteria significantly correlated with key root exudates, none but Actinobacteria were associated with the tobacco output. Considering the positive correlation of Actinobacteria to the major autotoxins, and the cancelling relationship between root exudates and tobacco output, we suggest that the root exudates are potential to indirectly affect the tobacco yield by affect bacterial community, and help to overcome the soil sickness through inhibiting the pernicious microbiota in the rhizosphere.

### Supplementary Information

Supplementary Figures.

Supplementary Tables.

## Data Availability

Raw sequences were deposited in the NCBI Sequence Read Archive with the accession number of PRJNA1035762 (http://www.ncbi.nlm.nih.gov/bioproject/1035762).
