# Supplementary material for: Root exudates and rhizosphere microbiota in responding to long-term continuous cropping of tobacco
Source: Sci Rep. 2024 May 17;14:11274. doi: 10.1038/s41598-024-61291-0 (PMC11101450; doi:10.1038/s41598-024-61291-0)

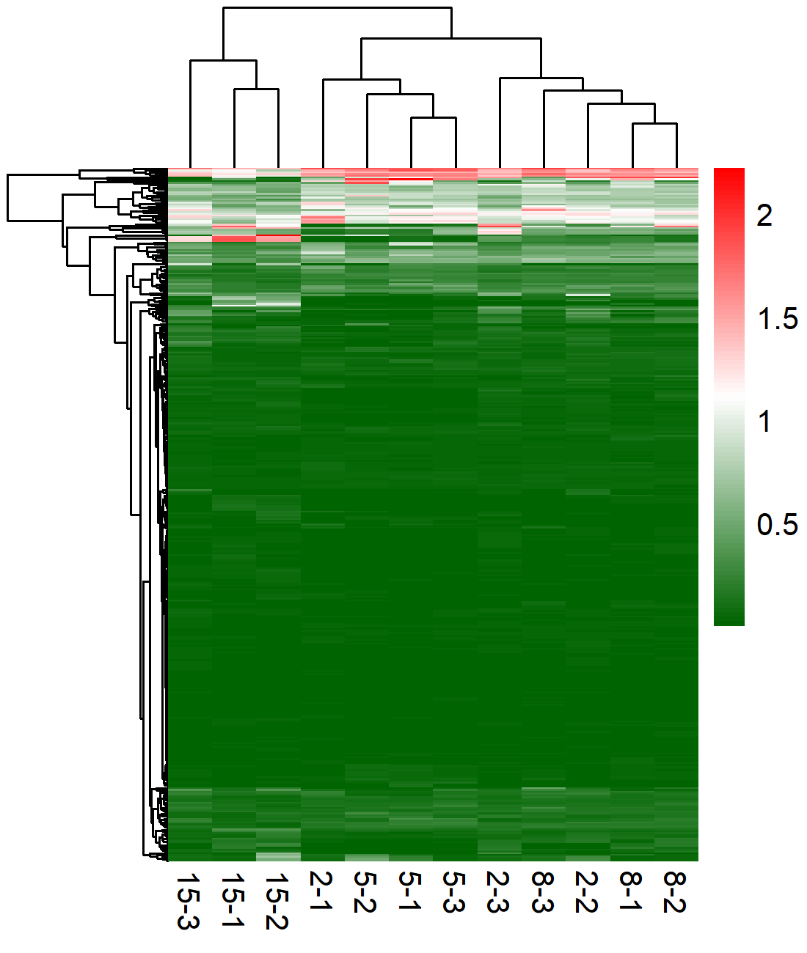
Figure S1 Heatmap reveals the difference in root exudate components of tobacco seedling planted in soils continuously cropping for 2, 5, 8 and 15 years.

Figure S2 Effect of long-term continuous cropping on the microbial community composition at genus level (a), and the correlation of dominate genus (b) to the key root exudates


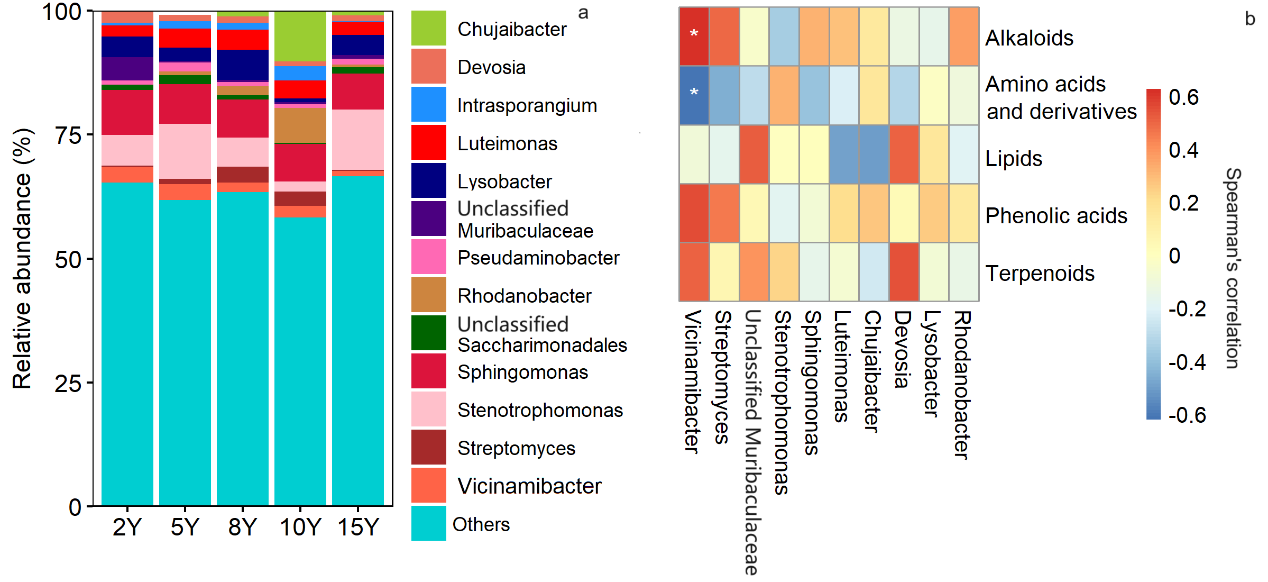

Supplement: Supplementary file 1 — Supplementary Figures. [file 41598_2024_61291_MOESM1_ESM.docx]
