# Supplementary material for: Root exudates and rhizosphere microbiota in responding to long-term continuous cropping of tobacco
Source: Sci Rep. 2024 May 17;14:11274. doi: 10.1038/s41598-024-61291-0 (PMC11101450; doi:10.1038/s41598-024-61291-0)
Supplement: Supplementary file 2 — Supplementary Tables. [file 41598_2024_61291_MOESM2_ESM.docx]

Table S1 Soil chemical properties after 2, 5, 8, 10 and 15 years (2Y, 5Y, 8Y, 10Y and 15Y) of continuous-cropping year. The analyzed soil properties including soil organic carbon (SOC), total nitrogen (TN), total phosphorus (TP), available nitrogen (AN), available phosphorus (AP) and available potassium (AK).

|  | SOC  （g kg^-^1） | TN  (g kg^-1^) | TP  (g kg^-1^) | AN  (mg kg^-1^) | AP  (mg kg^-1^) | AK  (mg kg^-1^) |
| --- | --- | --- | --- | --- | --- | --- |
| 2Y | 33.07 ^a^ | 1.91 ^b^ | 1.22 ^c^ | 18.69 ^c^ | 129.70 ^d^ | 91.93 ^d^ |
| 5Y | 32.36 ^a^ | 2.07 ^ab^ | 1.20 ^c^ | 20.87 ^b^ | 163.61 ^c^ | 99.33 ^c^ |
| 8Y | 32.47 ^a^ | 2.11 ^ab^ | 1.37 ^b^ | 21.19 ^b^ | 159.18 ^c^ | 101.97 ^c^ |
| 10Y | 31.05 ^a^ | 1.99 ^ab^ | 1.37 b | 19.86 ^bc^ | 249.15 ^b^ | 125.13 ^b^ |
| 15Y | 32.62 ^a^ | 2.35 ^a^ | 2.06 ^c^ | 23.95 ^a^ | 397.39 ^a^ | 196.47 ^a^ |

Table S2 Relationships of soil chemical properties to the tobacco yield and output value. The analyzed soil properties including soil organic carbon (SOC), total nitrogen (TN), total phosphorus (TP), available nitrogen (AN), available phosphorus (AP) and available potassium (AK).

|  |  | SOC | TN | TP | AN | AP | AK |
| --- | --- | --- | --- | --- | --- | --- | --- |
| Yield | R | 0.169 | 0.124 | 0.018 | 0.001 | 0.004 | 0.035 |
|  | *p* | 0.128 | 0.198 | 0.631 | 0.923 | 0.828 | 0.502 |
| Output value | R | 0.214 | 0.102 | 0.002 | 0.012 | 0.001 | 0.007 |
|  | *p* | 0.083 | 0.247 | 0.860 | 0.695 | 0.921 | 0.768 |
